# Increased Extracellular Osteopontin Levels in Normal Human Breast Tissue at High Risk of Developing Cancer and Its Association With Inflammatory Biomarkers *in situ*

**DOI:** 10.3389/fonc.2019.00746

**Published:** 2019-08-16

**Authors:** Gabriel Lindahl, Anna Rzepecka, Charlotta Dabrosin

**Affiliations:** ^1^Department of Oncology, Linköping University, Linköping, Sweden; ^2^Department of Clinical and Experimental Medicine, Linköping University, Linköping, Sweden; ^3^Department of Radiology, Linköping University, Linköping, Sweden; ^4^Department of Medical and Health Sciences, Linköping University, Linköping, Sweden

**Keywords:** inflammation, microdialysis, tamoxifen, flaxseed, enterolactone

## Abstract

Mammographic breast density is a strong independent risk factor for breast cancer (BC), but the molecular mechanisms behind this risk is yet undetermined and prevention strategies for these women are lacking. The anti-estrogen tamoxifen may reduce the risk of BC but this treatment is associated with severe side effects. Thus, other means for BC prevention, such as diet interventions, need to be developed. Osteopontin (OPN) is a major mediator of inflammation which is key in carcinogenesis. OPN may be cleaved by proteases in the tissue and cleaved OPN may in turn induce an inflammatory cascade in the extracellular microenvironment. We aimed to determine if extracellular OPN was altered in BC and in normal breast tissue with different densities and if tamoxifen or a diet of flaxseed could modify OPN levels. The study comprised 103 women; 13 diagnosed with BC, 42 healthy post-menopausal women with different breast densities at their mammography screen, and 34 post-menopausal women who added 25 g of ground flaxseed/day or were treated with tamoxifen 20 mg/day and were investigated before and after 6 weeks of exposure. Additionally, 10 premenopausal women who added flaxseed for one menstrual cycle and four who were investigated in two unexposed consecutive luteal phases of the menstrual cycle. Microdialysis was used to sample extracellular proteins *in vivo* in breast tissue and proteins were quantified using a multiplex proximity extension assay. We found that, similar to BC, extracellular *in vivo* OPN levels were significantly increased in dense breast tissue. Additionally, significant correlations were found between OPN and chemokine (C-X-C motif) ligand (CXCL)-1, −8, −9, −10, and −11, interleukin-6, vascular endothelial growth factor, matrix metalloproteinase (MMP)-1, −2, −3, 7, and −12 and urokinase-type plasminogen activator whereas no correlations were found with MMP-9, chemokine (C-C motif) ligand (CCL)-2, and −5. Estradiol did not affect OPN levels in breast tissue. None of the interventions altered OPN levels. The pro-tumorigenic protein OPN may indeed be a molecular target for BC prevention in women with increased breast density but other means than tamoxifen or flaxseed i.e., more potent anti-inflammatory approaches, need to be evaluated for this purpose.

## Introduction

Osteopontin (OPN), is an extracellular secreted protein produced by many different cell types in the body (1). OPN was initially identified as extracellular matrix protein in bone, bone sialprotein 1, and thereafter as a secreted protein from several cancer cells culture (2, 3). In normal tissues, OPN is involved in several pathophysiological functions such as vascular and bone remodeling, wound repair, and inflammation (4). OPN is an intergrin-binding protein, key in the inflammatory response, and thus up-regulated in inflammatory conditions as well as in several cancer forms including breast cancer (4–7). Immunohistochemistry studies have also suggested an association between a spliced isoform of OPN, and increased risk of invasiveness in premalignant breast lesions (8). However, in breast cancer patients, no correlations between circulating plasma levels of OPN and staining intensity of cellular OPN in breast cancers have been determined (9). It has been suggested that it is the secreted OPN, locally in the tissue, that is necessary for indolent cancer cells to develop metastases (10). The biological activity of OPN can be modulated by proteolytic cleavage in the microenvironment and has been shown to be a substrate for several matrix metalloproteinases (MMPs) including MMP-2, −3, −7, and −9 (11–13). This cleaved OPN may enhance invasion and metastases formation of cancer cells (11–13). Indeed, this emphasizes the need of local sampling of OPN, within the target organ, for elucidations of its role in pathophysiological processes.

OPN acts as a chemoattractant to macrophages, and may also affect the phenotypically skewing of these cells (14). Furthermore, OPN may act as a chemotactic for neutrophils, dendritic cells, NK-cell, and polarize T-cells by interacting with CD44 (15). In experimental models, OPN expression increased the pro-inflammatory cytokine IL-6 and these findings has been corroborated in humans (16–18). Additionally, an interconnection and correlation has been determined between these two proteins, and increased levels have been associated with increased metastatic spread and poor prognosis in cancer patients (18, 19). Several other cytokines and chemokines important for the immune response and chemotaxis of inflammatory cells such as chemokine (C-C motif) ligands (CCLs) and chemokine (C-X-C motif) ligands (CXCLs) have also been associated with OPN both in cancer and inflammatory conditions (20–22). OPN may also play an important role in regulating angiogenesis by autocrine and paracrine regulation of vascular endothelial growth factor (VEGF) in several experimental cancer forms including breast cancer (23–25).

Mammographic density i.e., the amount of radiological opaque tissue as compared to fat tissue in the breast, is a major independent risk factor for breast cancer and represents at least a 4-fold increased risk (26, 27). The sensitivity of detecting breast cancers in dense breast tissue may be compromised but it has been shown that the increased risk cannot be explained by this “masking” effect (28). Less than 10% of normal breast tissue comprise epithelial cells and conflicting data regarding the amount and proliferation rate of these cells in dense vs. non-dense normal breast has been reported (29–33). The major difference of these two tissue types is the stroma; dense breast tissue contains higher amounts of stroma, including collagen, and non-dense breasts contain higher amounts of fat tissue (31, 32). However, the biological mechanisms underlying the increased risk of breast cancer in dense breasts are poorly understood. Exposure sex steroids including estrogens is an established risk factor for breast cancer (34, 35) but an associations between circulating estrogen levels and breast density is lacking (26).

Active biological pathways in dense breast tissue need to be unraveled in order to develop effective preventive therapeutics against breast cancer for this group of women. The role of OPN in breast tissue at high risk of developing breast cancer is unexplored.

Here, we investigated levels of OPN and its interconnection *in vivo* with inflammatory and angiogenic proteins and MMPs in human breast cancer, normal human breast tissue, and after interventions with the anti-estrogen tamoxifen or diet addition of flaxseed. We show that the extracellular *in vivo* levels of OPN were significantly increased in breast cancers and dense breast tissue as compared to their normal counterparts. In normal breast tissue, strong correlations were found between OPN and several pro-inflammatory mediators. Our data did not support an estrogen dependent regulation of OPN and no effects of tamoxifen or addition of dietary flaxseed on OPN levels in normal breast tissue were detected. Our data suggest that OPN may indeed be a therapeutic target for prevention in women with dense breast tissue but other means than tamoxifen and flaxseed need to be developed.

## Materials and Methods

### Subjects

The Regional Ethical Review Board of Linköping, Sweden, approved the study, which was carried out in accordance with the Declaration of Helsinki. All subjects gave written informed consent. A total of 103 women were included in the study. Thirteen post-menopausal women who were diagnosed with breast cancer were investigated before surgery. Forty-two healthy post-menopausal women were consecutively recruited from the mammography screening program at Linköping University Hospital. Their regular screening mammograms were categorized as either entirely fatty non-dense or extremely dense according to the Breast Imaging Reporting and Data System (BI-RADS) density scale; women with BI-RADS A (non-dense) or BI-RADS D (dense) were selected (36). Post-menopausal women with previous ER-positive early breast cancer that had been surgically removed and were advised tamoxifen 20 mg/day as adjuvant therapy were investigated before (*n* = 21) and after 6 weeks of treatment (*n* = 19), two women were omitted from the second microdialysis investigation because of non-compliance. Additionally, 27 healthy volunteers were included for the diet intervention; 13 post-menopausal women added 25 g of ground flaxseed/day were investigated before start, and after 6 weeks of diet addition, 14 women were premenopausal and investigated in two consecutive luteal phases out of which 10 added flaxseed, as described above, for one menstrual cycle and as a control four were investigated in two unexposed consecutive luteal phases. All of the premenopausal women had a history of regular menstrual cycles (cycle length, 27–34 d). None of the healthy women had a history of previous breast cancer. In addition, none of the women were currently using (or had used within the previous 3 months) hormone replacement therapy (HRT), sex steroid-containing contraceptives, anti-estrogen therapies, including selective estrogen receptor modulators or degraders or antibiotics within past 3 months.

### Microdialysis Procedure

Prior insertion, 0.5 ml lidocain [10 mg*/*mL] was administrated intracutaneously. Microdialysis catheters (71*/*M Dialysis AB, Stockholm, Sweden), which consists of a tubular dialysis membrane (diameter 0.52 mm, 100,000 atomic mass cut-off) glued to the end of a double-lumen tube were inserted via a splitable introducer (M Dialysis AB), connected to a microinfusion pump (M Dialysis AB) and perfused with NaCl 154 mmol*/*L and hydroxyethyl starch 60 g/L (Voluven®, Fresenius Kabi, Uppsala, Sweden), at 0.5 μL/min. The women with ongoing breast cancer were investigated with a 10 mm membrane before surgery; one catheter was inserted within the cancer and the other in normal adjacent breast tissue. All other women were investigated with a 20 mm long microdialysis membrane; in healthy volunteer women the microdialysis catheter was placed in the upper lateral quadrant of the left breast directed toward the nipple and in the women with previous breast cancer the catheter was inserted in the upper lateral quadrant of the unaffected breast as previously described (37–47).

After a 60-min equilibration period, the outgoing perfusate was stored at −80°C for subsequent analysis.

The recovery of compounds from the extracellular space over the microdialysis membrane is dependent on the surface area of the membrane. Therefore, quantitative comparisons can only be performed on data retrieved from membranes of equal sizes; breast cancer vs. normal adjacent breast tissue (10 mm membrane), or normal breast tissue from all other groups (20 mm membrane).

### Protein Quantifications

The microdialysis samples were analyzed by using a multiplex proximity extension assay (PEA, Olink Bioscience, Uppsala Sweden). In brief, 1 μL sample was incubated in the presence of proximity antibody pairs tagged with DNA reporter molecules. Once the pair of antibodies is bound to their corresponding antigens, the respective DNA tails form an amplicon by proximity extension, which was quantified by high-throughput real-time PCR (BioMark™ HD System, Fluidigm Corporation). The generated fluorescent signal directly correlates with protein abundance. The output from the Proseek Multiplex protocol is in quantitation cycles (Cq) produced by the BioMark's Real-Time PCR Software. To minimize variation within and between runs, the data are normalized using both an internal control (extension control) and an interplate control, and then transformed using a pre-determined correction factor. The pre-processed data were provided in the arbitrary unit normalized protein expression (NPX) on a log_2_ scale, which were then linearized by using the formula 2^NPX^. A high NPX value corresponds to a high protein concentration. Values represent a relative quantification meaning that no comparison of absolute levels between different proteins can be made.

### Estradiol and Enterolactone Determinations

Serum was analyzed with immunoassay kits based on the principle of competitive binding. The kits were used according to the manufacturer's instructions. For estradiol an ELISA immunoassay kit (Calbiotech Spring Valley, CA) was used and for enterolactone using time-resolved fluoroimmunoassay (Labmaster TR-FIA, Turku, Finland). This method has been shown to have a significant linear relationship with other techniques such as GC-MS and LC-MS (48, 49).

### Statistical Analyses

Statistical analyses were performed using non-parametric Wilcoxon matched-pairs signed rank test or unpaired Mann-Whitney *U*-test. The data was non-normally distributed and therefore Pearson's correlations coefficient was computed on ranked data. All tests were two-sided. A *p* < 0.05 was considered as statistically significant. Statistics were performed with Prism 7.0 (GraphPad software).

## Results

In Table 1, routine determinations of tumor histology, size, immunohistochemistry for estrogen receptor (ER) and progesterone receptor (PR), HER-2 receptor, and Nottingham histological grade (NHG) according to the Elston Ellis scoring system are shown. There were no subsequent complications after the microdialysis investigations. Eleven of the 13 patients were ER+ with an ER score >50%. The breast density of the women with ongoing breast cancer was not known.

**Table 1:** Characteristics of patients subjected to intratumoral microdialysis.

**Patient**	**Age**	**Tumor size**	**Grade (NHG)**	**ER (%)**	**PR (%)**	**HER-2**
1	70	22	2	>50	>50	Neg
2	68	24	2	>50	>50	Neg
3	52	25	3	>50	10–50	Neg
4	78	28	2	>50	>50	Neg
5	62	25	2	>50	>50	Neg
6	63	19	2	>50	>50	Neg
7	55	40	3	0	0	Neg
8	61	25	2	>50	>50	Neg
9	48	30	2	>50	>50	Neg
10	73	30	2	>50	<5	Neg
11	51	27	1	>50	>50	Neg
12	66	60	2	>50	>50	Neg
13	80	50	ND	0	0	Pos

*ER, estrogen receptor, PR = progesterone receptor; NHG, Nottingham histological grade; HER-2, human epidermal growth factor receptor 2; ND, not determined*.

BMI of the 103 women was within the normal range 21–30 except for one woman in the tamoxifen group (BMI 36), one women in post-menopausal flax group (BMI 32), and one woman in the non-dense group (BMI of 32).

### Extracellular OPN Was Significantly Increased in Breast Cancer and Dense Breast Tissue

**Figure 1:**
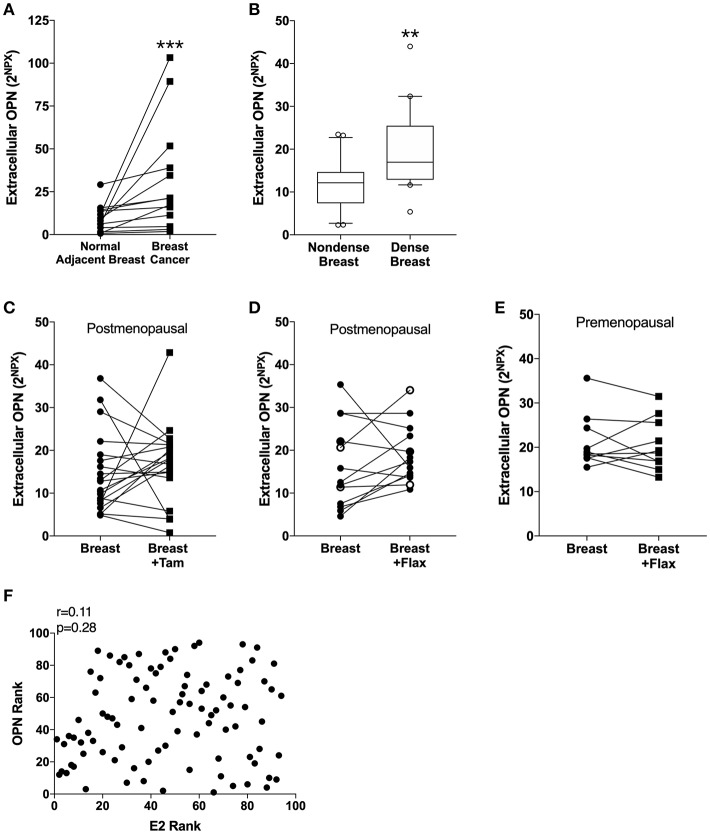
Extracellular levels of Osteopontin (OPN) in breast tissue *in vivo*. Microdialysis was used to sample *in vivo* extracellular molecules from breast tissue of women. Proteins were quantified using a multiplex proximity extension assay as described in the materials and methods section. **(A)** OPN in breast cancer. Thirteen breast cancer patients underwent microdialysis before surgery. One catheter was inserted into the breast cancer and another into adjacent normal breast tissue. **(B)** OPN in breast tissue of post-menopausal women with various mammographic density. Forty post-menopausal healthy volunteer women, attending the regular mammography-screening program and were categorized as either having dense (*n* = 20) or non-dense (*n* = 20) breasts underwent microdialysis of their left breast. Boxplots with median and 10–90 percentile are depicted. **(C)** OPN in normal breast tissue before and after tamoxifen treatment. Nineteen post-menopausal women were investigated in their unaffected normal breast before and after 6 weeks of adjuvant tamoxifen therapy 20 mg/day. **(D)** OPN in normal breast tissue after flaxseed ingestions in post-menopausal women. Thirteen post-menopausal healthy volunteer women were investigated in their left breast before and after 6 weeks of a diet addition of 25 mg ground flaxseed/day. The open symbols represent the two women that did not convert the flaxseed into entereolactone. **(E)** OPN in normal breast tissue after flaxseed ingestions in premenopausal women. Ten premenopausal healthy volunteer women were investigated in their left breast before and after a diet addition of 25 mg ground flaxseed/day for one menstrual cycle. All participants converted flaxseed into entereolactone. In **(A–E)**; Wilcoxon matched-pairs signed rank test was used for paired data and Mann-Whitney *U*-test for unpaired data. ^**^P < 0.01 and ^***^P < 0.001. **(F)** *No correlation between OPN in breast tissue and estradiol (E2) levels*. Microdialysis was used to sample *in vivo* extracellular molecules from breast tissue of women. Proteins were quantified using a multiplex proximity extension assay and estradiol with a competitive ELISA as described in the materials and methods section. A total of 94 microdialysis investigations of normal breast tissue unexposed to any treatment is depicted; 42 post-menopausal healthy volunteer women, attending the regular mammography-screening program; 21 post-menopausal women treated for early breast cancer investigated in their unaffected normal breast before the start of adjuvant tamoxifen therapy; 13 post-menopausal healthy volunteer women before the start of dietary flaxseed addition; 10 premenopausal healthy volunteer women before the start of dietary flaxseed addition and four premenopausal women in two consecutive luteal phases of the menstrual cycle. Statistics were calculated using Pearson's correlations coefficient on ranked data.

In line with previous data of circulating OPN in blood from breast cancer patients, the extracellular *in situ* levels of OPN in breast cancers compared with normal adjacent breast tissue were significantly increased (Figure 1A).

Of the 42 women recruited from the mammography screening program 21 were initially categorized as having dense breasts and 21 with non-dense breasts. After re-evaluation of the mammograms one woman in each group had to be excluded because of miss-categorization. In dense breast tissue (*n* = 20), as compared to non-dense breast tissue (*n* = 20), OPN exhibited significantly increased levels (Figure 1B).

### Tamoxifen Treatment and Addition of Dietary Flaxseed Did Not Alter OPN Levels

OPN has previously been shown to be an estrogen regulated gene in rodents (50, 51). Additionally, phytoestrogens have also reported to affect OPN levels in murine prostate cancer (52, 53). Therefore, we wanted to explore if tamoxifen treatment or a diet addition of flaxseed, which is converted to the phytoestrogen enterolactone by the gut microbiota, affected the local levels of OPN in breast tissue. Contrary to the experimental data the anti-estrogen tamoxifen did not did not alter the levels of OPN in normal breast tissue in post-menopausal women (Figure 1C). Similarly, a diet addition with flaxseed had no effect on OPN levels in the breast in post-menopausal women (Figure 1D). As there may be a difference in the response to dietary flaxseed between pre- and post-menopausal women this was also tested in a premenopausal cohort. However, diet addition of flaxseed to premenopausal women did not affect the OPN levels in breast tissue (Figure 1E). No correlation between estradiol levels and OPN in normal breast tissue was found supporting the lack of effects by the different anti-estrogen approaches, *n* = 94, *r* = 0.11, *p* = 0.28 (Figure 1F).

### Extracellular *in vivo* OPN and Extracellular *in vivo* MMP-1, −2, −3, −7, and −12 and uPA in Normal Breast Tissue Correlated Significantly

**Figure 2:**
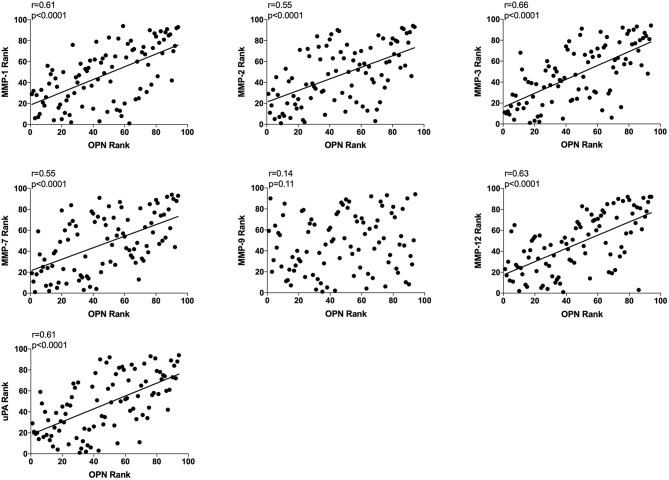
Extracellular breast Osteopontin (OPN) levels *in vivo* and its correlations with extracellular protease levels *in vivo* in normal human breast tissue. Microdialysis was used to sample *in vivo* extracellular molecules from breast tissue of women. Proteins were quantified using a multiplex proximity extension assay as described in the materials and methods section. A total of 94 microdialysis investigations of normal breast tissue unexposed to any treatment is depicted; 42 post-menopausal healthy volunteer women, attending the regular mammography-screening program; 21 post-menopausal women treated for early breast cancer investigated in their unaffected normal breast before the start of adjuvant tamoxifen therapy; 13 post-menopausal healthy volunteer women before the start of dietary flaxseed addition; 10 premenopausal healthy volunteer women before the start of dietary flaxseed addition; and four premenopausal women in two consecutive luteal phases of the menstrual cycle. Statistics were calculated using Pearson's correlations coefficient on ranked data.

All data from unexposed normal breast tissue i.e., including data from the tamoxifen and flaxseed cohorts before start of treatments, were included in the correlation analyses, *n* = 94. A significant positive correlation was found between local extracellular *in vivo* levels in normal breast tissue between OPN and MMP-1, −2, −3, −7, and −12, and uPA whereas no correlation was detected between OPN and MMP-9 (Figure 2).

Subgroup analyses are included in Table 2. However, because of the limited sample size in each subgroup these data should be interpreted with caution.

**Table 2:** Subgroup analysis of the correlations between osteopontin and MMPs (matrix metalloproteinases), uPA (urokinase-type plasminogen activator), interleukin (IL), CXCL (chemokine (C-X-C motif) ligand), and CCL (Chemokine (C-C motif) ligand) in normal human breast tissue *in vivo*.

	**Dense** ***n* = 20**	**Non-dense** ***n* = 20**	**Before tamoxifen** ***n* = 21**	**Post-menopausal before flax** ***n* = 13**	**Premenopausal before flax** ***n* = 18**
MMP-1	**0.49**	**0.67**	**0.54**	**0.71**	0.25
MMP-2	**0.52**	**0.40**	**0.69**	**0.59**	**0.42**
MMP-3	**0.46**	**0.52**	**0.68**	**0.67**	0.01
MMP-7	**0.40**	0.13	**0.73**	**0.70**	**0.56**
MMP-9	−0.25	0.09	−0.24	0.00	−0.22
MMP-12	**0.56**	**0.62**	**0.56**	**0.80**	0.01
uPA	**0.42**	**0.53**	**0.62**	**0.69**	−0.01
IL-6	0.21	0.34	0.19	**0.42**	**0.46**
CXCL1	0.27	**0.58**	0.20	−0.15	0.04
CXCL8	0.15	**0.53**	−0.04	−0.53	0.02
CXCL9	**0.54**	**0.37**	**0.72**	**0.83**	**0.56**
CXCL10	**0.49**	0.33	**0.67**	**0.42**	**0.66**
CXCL11	0.13	**0.50**	0.18	**0.65**	**0.41**
CCL2	0.18	**0.37**	0.09	−0.14	−0.02
CCL5	−0.05	0.19	0.20	0.29	**−0.42**
VEGF	**0.69**	**0.66**	0.20	**0.87**	−0.14

*Spearman's correlation r. Significant values in bold p < 0.05*.

### Extracellular *in vivo* OPN and Extracellular *in vivo* IL-6, CXCL-1, −8, −9, −10, −11, and VEGF in Normal Breast Tissue Correlated Significantly

**Figure 3:**
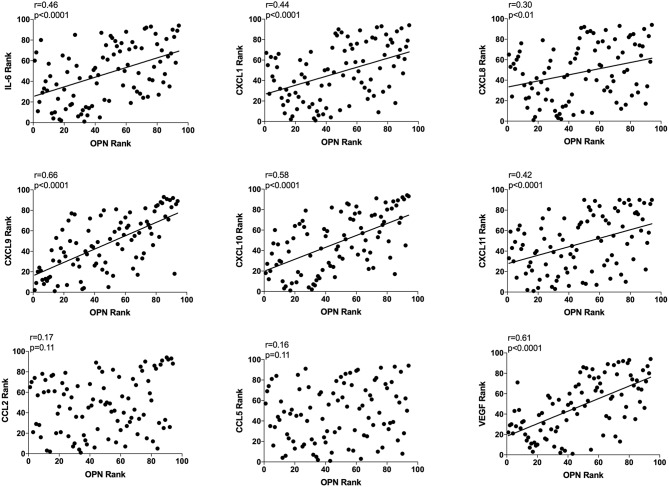
Extracellular breast Osteopontin (OPN) levels *in vivo* and its correlations with extracellular inflammatory- and angiogenic proteins *in vivo* in normal human breast tissue. Microdialysis was used to sample *in vivo* extracellular molecules from breast tissue of women. Proteins were quantified using a multiplex proximity extension assay as described in the materials and methods section. A total of 94 microdialysis investigations of normal breast tissue unexposed to any treatment is depicted; 42 post-menopausal healthy volunteer women, attending the regular mammography-screening program; 21 post-menopausal women treated for early breast cancer investigated in their unaffected normal breast before the start of adjuvant tamoxifen therapy; 13 post-menopausal healthy volunteer women before the start of dietary flaxseed addition; 10 premenopausal healthy volunteer women before the start of dietary flaxseed addition and four premenopausal women in two consecutive luteal phases of the menstrual cycle. Statistics were calculated using Pearson's correlations coefficient on ranked data.

As OPN is a potent regulator of inflammation and angiogenesis we also determined correlations with key potent proteins of these events, *n* = 94. OPN exhibited a significant positive correlation with IL-6, CXCL-1, −8, −9, −10, −11, and VEGF as shown in Figure 3. CXCL-9, −10, and VEGF exhibited higher r values in the relationship with OPN compared to all other proteins (Figure 3). No correlations were found between OPN and CCL-2 or CCL-5 (Figure 3).

Subgroup analyses are included in Table 2. However, because of the limited sample size in each subgroup these data should be interpreted with caution.

### Extracellular *in vivo* OPN and Correlations in Breast Cancers

Next, we investigated if there were any correlations between OPN and extracellular proteins in breast cancers. As the 13 breast cancers included in the study had a diverse biology, correlations may be difficult to detect because of the sample size and data should be interpreted with caution. Yet, significantly positive correlations were found between OPN and MMP-1 *r* = 0.78, *p* < 0.01, MMP-2, *r* = 0.55, *p* < 0.05, MMP-3, *r* = 0.62, *p* < 0.05, and MMP-12, *r* = 0.87, *p* < 0.0001 whereas no correlations were found with MMP-7 and −9 or uPA. OPN also correlated significantly with CXCL-9, *r* = 0.89, *p* < 0.001 whereas no correlations were found with IL-6, CXCL-1, −8. 10, and −11 or CCL-2, −5, and VEGF.

## Discussion

Findings herein demonstrate significantly increased extracellular *in vivo* levels of OPN in breast cancers of women. Our data also revealed that, similar to breast cancers, normal dense breast tissue, with an intrinsically very high risk of developing breast cancer, exhibited significantly increased OPN levels as compared to low risk non-dense breast tissue.

In normal breast tissue OPN levels correlated significantly with MMP-1, −2, −3, −7, and −12 and uPA but not with MMP-9. OPN also correlated with VEGF, IL-6, CXCL-1, −8, −9, −10, and −11 but not with CCL-2 and CCL-5. Therapeutic interventions with tamoxifen or dietary addition of flaxseed to pre- and post-menopausal women did not alter the OPN levels in normal breast tissue. No correlation between estradiol and OPN was detected supporting the results of the anti-estrogen interventions.

More than 50% of the body weight consists of body fluid and approximately one third of this fluid can be found outside the cells in tissues. This interstitial or extracellular fluid contain a reservoir of molecules released by the different cell types in the tissue, controlling pathophysiological processes in the microenvironment. This major component in the body is, however, still relatively unexplored and needs to be characterized in order to understand pathophysiology. Paracrine signaling, and thus the homeostasis of the microenvironment, is regulated by soluble factors in the interstitial fluid and one major difficulty in studying this, is retrieval of molecules from this compartment. One major strength of this study is therefore our *in vivo* approach for sampling of extracellular molecules using microdialysis directly in live tissue *in situ*. This allows for determinations of previously unrecognized metabolic events.

To the best or our knowledge, extracellular OPN *in vivo* has previously not been determined in human breast tissues including breast cancer before. We found increased extracellular OPN levels in breast cancers, which is in keeping with previous studies that have shown increased plasma levels of OPN in metastatic breast cancer patients (54, 55). OPN levels have also been associated with tumor burden, lymph node metastases, and poor survival (9, 54–56). In the adjuvant setting, however, OPN measured in plasma has not been shown to be prognostic in multivariate analyses (9). Plasma OPN do not necessarily reflect breast tissue levels as all cells in the body contribute to these plasma levels. Our data of increased levels of OPN at its bioactive site, directly in the cancerous tissue indeed confirms that extracellular OPN may play a key role in breast cancer biology.

OPN plays a significant role in tumor progression by shaping the cancer microenvironment (6, 11–16, 19, 21). Our data, with increased levels in dense breast tissue, clearly suggests that local OPN may also be up-regulated in non-cancerous tissues. Additionally, OPN exhibited significant positive correlations with several proteases in normal breast tissue. Proteases mediate a continuous remodeling of the extracellular matrix and have been shown to have a broad range of substrates including several cytokines. Protease expression has been implicated to be essential in cancer progression. However, clinical trials of broad-spectrum MMP inhibitors have failed and even shown an increased risk of tumor progression (57–59). Clearly, some MMPs may have anti-tumor effects and possible future interventions against MMPs must be more selective (59). Interestingly, MMP-9, which failed to show any correlation with OPN in our data-set, has been shown to be anti-tumorigenic in experimental cancer models by affecting levels of anti-angiogenic fragments and inflammation (60–62). Additionally, in human breast cancers no increased levels of extracellular MMP-9 has been detected (63). Our present data of an interconnections between OPN and other proteases such as MMP-1, −2, −3, −7, −12, and uPA, in normal human breast tissue are in line with previous studies of several cancer forms (11–13).

We also found that OPN exhibited significant correlations with several inflammatory and angiogenic proteins in normal breast tissue. Some of these correlations corroborate previous findings from cancerous tissues such as IL-6 and VEGF whereas others do not, including CCL-2 and −5. Nevertheless, several of these alternations seems to be unfavorable regarding carcinogenesis.

OPN may mediate its effect through several different mechanisms though some need yet to be elucidated (15). However, it has been shown that OPN activates the phosphatidylinositol 3-kinases/Protein kinase B/nuclear factor kappa B (PI3K/AKT/NFKB) pathway inducing uPA secretion and MMP-2 activation in breast cancer cells (64). It has also been shown that both the PI3K/AKT and extracellular signal–regulated kinases 1 and 2 (ERK 1/2) pathways are involved in the OPN induced secretion of VEGF in endothelial cells (65).

The OPN gene has been suggested to be under the control of estrogens, at least in rodent models (50, 51). In our data-set estradiol failed to relate to OPN levels. One strength of our data is that both premenopausal women with high levels of estradiol as well as post-menopausal women with considerably lower estradiol levels were included. Despite this we were not able to find any evidence that estradiol affects OPN in normal human breast tissue. Moreover, OPN levels were unaffected by treatment with the anti-estrogen tamoxifen as well as by a diet addition of flaxseed. Together, these data strongly suggest that OPN in normal human breast tissue is under the control of other pathways than that of estrogen.

Non-toxic potent breast cancer prevention would obviously be the most effective strategy for decreasing mortality and morbidity of breast cancer. Today, such prevention is not available. The anti-estrogen tamoxifen is registered in some countries as a breast cancer prevention therapy. Tamoxifen and aromatase inhibitors to women at high risk of developing breast cancer has been shown to reduce the risk of breast cancer by 30–50% (66–68). This treatment is associated with severe side-effects such as thromboembolism, endometrial cancer, osteoporosis, and low quality of life leading to a very low compliance to the therapy (66, 67). Thus, other efficient non-toxic breast cancer prevention therapeutics, possible to comply to for a long period of time, are needed. Diet may be one such intervention as epidemiological and migrant studies indicate that Asian population have decreased risk of breast cancer depending on life style factors (69). Exposure to phytoestrogens such as genistein and enterolactone may be one explanation and in mice the genistein has been shown to affect OPN in prostate cancer (52, 53). In Western diets, lignans including enterolactone is the major ingested phytoestrogen. Flaxseed is one major source of enterolignans and we have shown that dietary flaxseed decreases pro-angiogenic proteins *in vivo* (37, 70). Flaxseed may be converted by the gut microbiota into enterolactone but not all individuals have this capacity (71). Two women that added flaxseed to the diet in our present study did not increase their enterolactone levels. However, our data, with or without these two women, and both in pre- and post-menopausal women did not indicate any change of OPN in breast tissue by the flaxseed diet. Clearly, other therapeutics need to be used for targeting OPN in the breast.

In conclusion, we demonstrate that extracellular OPN was increased in human breast cancers *in vivo*. Similar to breast cancer, normal dense breast tissue in post-menopausal women exhibited significantly higher levels of OPN than non-dense breast tissue. In normal breast tissue OPN was associated with several pro-tumorigenic proteins, which may enhance a cancer-permissive microenvironment. As OPN, irrespective of its source, is a pro-tumorigenic protein it seems desirable to target this molecule in prevention strategies. However, OPN levels in normal breast tissue was not modifiable with tamoxifen or a diet addition of flaxseed and estradiol did not affect the levels. Thus, other therapeutics targeting OPN may be more feasible to include in breast cancer preventions trials of women with dense breast tissue.

## Data Availability

All datasets generated for this study are included in the manuscript and/or the supplementary files.

## Ethics Statement

The Regional Ethical Review Board of Linköping, Sweden, approved the study, which was carried out in accordance with the Declaration of Helsinki. All subjects gave written informed consent.

## Author Contributions

GL and CD collaborated on the study conception, study design, interpretation of data, performed the data analysis, and drafted the manuscript. CD performed all microdialysis experiments. AR assed the mammographic density. All authors have read and approved the final manuscript.

### Conflict of Interest Statement

The authors declare that the research was conducted in the absence of any commercial or financial relationships that could be construed as a potential conflict of interest.
